# Prevalence of lactose intolerance in patients with diarrhea-predominant irritable bowel syndrome: data from a tertiary center in southern China

**DOI:** 10.1186/s41043-017-0113-1

**Published:** 2017-11-21

**Authors:** Lishou Xiong, Yilin Wang, Xiaorong Gong, Minhu Chen

**Affiliations:** 1 Department of Gastroenterology, The First Affiliated Hospital, Sun Yat-Sen University, 58 Zhongshan II Road, Guangzhou, 510080 People’s Republic of China; 2 Department of Gastroenterology, The First People’s Hospital of Foshan, Foshan, 528000 People’s Republic of China

**Keywords:** Irritable bowel syndrome, Lactose intolerance, Breath test

## Abstract

**Background:**

Symptoms associated with lactose intolerance (LI) and diarrhea-predominant irritable bowel syndrome (IBS-D) are almost the same. These disease entities are difficult to differentiate clinically. In practice, differential diagnosis depends on self-reported patient milk intolerance. However, there is limited data on the prevalence of LI in China. The aim of this study was to investigate the prevalence of LI in IBS-D patients and asymptomatic healthy controls.

**Methods:**

Lactose malabsorption (LM) was diagnosed by a lactose hydrogen breath test (HBT) and was defined by peak breath H_2_ excretion over the baseline level of ≥ 20 ppm. LI-related symptoms were monitored for 8 h following lactose administration. LI was defined in LM patients with positive symptoms during the observation time. Patients with IBS-D were additionally asked if they were intolerant to milk.

**Results:**

A total of 109 eligible IBS-D patients (Rome III criteria) and 50 healthy controls were enrolled in this study. Except for hydrogen non-producers, the prevalence of LM did not differ between IBS-D patients and the control group (85%, 82/96 vs 72%, 34/47; *P* = 0.061). There was, however, a higher LI prevalence in IBS patients than in healthy subjects (45%, 43/96 vs 17%, 8/47; *P* = 0.002). Sensitivity, specificity, and positive and negative predictive values of self-reported milk intolerance for detecting LI were 58, 58, 53, and 63%, respectively.

**Conclusions:**

Prevalence of LI is significantly higher in IBS-D patients than in healthy subjects. Self-reported milk intolerance is a poor diagnostic predictor of LI.

## Background

Irritable bowel syndrome (IBS) is a common and global gastrointestinal disorder, and the pathogenesis of IBS is unclear [1]. Diarrhea-predominant IBS (IBS-D), a subtype of IBS which can present some similar symptoms with lactose intolerance (LI) such as abdominal pain, bloating, flatulence, and diarrhea, hampers differentiation between disease types [2]. The prevalence of LI in patients with IBS is unclear. Many studies show that the frequency of LI is higher in patients with IBS than that in healthy individuals [3–5]. Some studies reported the comparable LI prevalence in these two groups [6]. In most circumstances, the concept of LI and lactose malabsorption (LM) is confusing and interchangeable. LM does not necessarily result in the development of intolerance symptoms, and only about one third to half of patients with LM are LI positive. The confusion of LM and LI hampers the interpretation of published data [7, 8]. In addition, previous studies focused on LI did not subtype IBS into IBS-D and constipation-predominant IBS (IBS-C) in that IBS-C has little correlation with LI. There is an obvious requirement for high-quality data on the prevalence of LI in IBS-D. It is especially true for Asian populations, where LI is thought to be common [9]. But there is very little data associated with LI in Chinese patients with IBS-D and information about this relationship is warranted.

A further consideration here is that physicians often rely on patients’ self-reporting of milk intolerance to diagnose LI. Several recent studies have shown that self-reported milk intolerance in IBS patients is not reliable in predicting the outcome of a LI test [4, 10].Thus, further studies employing validated tools for diagnosing LI are necessary.

The aims of this study were to evaluate the frequency of LM and LI in patients with IBS and compare these results with the asymptomatic population of southern China. Furthermore, we assessed the performance of self-reported milk intolerance for IBS-D patients for detecting bona fide LI.

## Methods

### Study participants

Consecutive outpatients diagnosed with IBS-D using the “Rome III criteria” in the Department of Gastroenterology at the First Affiliated Hospital of Sun Yat-Sen University (Guangdong Province, China) were enrolled in this study. Sex-matched healthy subjects were also enrolled in the same study. The age range of the selected individuals was 18 to 65 years of age. Exclusion criteria included “alarm symptoms” (weight loss in the previous year, blood in stools, and colorectal cancer in first-degree relatives), ingested antibiotics, proton pump inhibitors, probiotics or any other drugs that affect gastric empting within 1 month before inclusion in the study, and any test that required cleansing of the bowel within 1 week of the study. None of the patients enrolled in this study had liver disease, chronic pancreatitis, diabetes, hypothyroidism, human immunodeficiency virus, or a history of abdominal surgery (except appendectomy or cesarean section).

All patients with IBS-D were classified as self-reported milk tolerant/intolerant according to patients’ individual interpretation. The study was approved by the ethical committee of The First Affiliated Hospital of Sun Yat-Sen University, and all participants signed informed consent forms prior to enrollment.

### Lactose hydrogen breath test (HBT)

Lactose HBT was performed using a gas analyzer (GastroLyzer® Breath Hydrogen Monitor; Bedfont Science Ltd., UK). The evening before the test was performed, subjects were asked to avoid foods containing complex carbohydrates (bread, potato, and corn) and fiber, in order to minimize basal hydrogen excretion. Patients were required to fast for at least 12 h before the breath test. Cigarette smoking and physical exercise were prohibited for 2 h before and during the test. Subjects brushed their teeth in the morning and gargled with 20 ml of chlorhexidine mouthwash (Koutai, Shenzhen, China) to eliminate fermentation by oropharyngeal bacteria flora [11].

Baseline hydrogen breath levels were obtained, and patients who had high values (greater than 20 ppm) were excluded from the study. Thirty seconds after determination of baseline hydrogen breath levels, subjects ingested 25 g of lactose dissolved in 100 ml water. Thereafter, lactose HBT was determined every 15 min for a duration of 3 h. An abnormally high measurement in the lactose HBT (peak hydrogen breath excretion of 20 ppm above the baseline level within 3 h of lactose ingestion) was used to identify the patient as LM positive [12]. Symptoms, such as abdominal pain, bloating, borborygmi, and diarrhea during the 8-h interval following the test, were recorded. Symptoms were graded according to a visual analog scale in which 0 = absence, 1 = trivial, 2 = mild, 3 = moderate, 4 = strong, and 5 = severe. Patients with LM were diagnosed as being LI positive when more than one time point displayed an increase in symptom severity. This diagnosis was based on at least two of the predetermined symptoms during the observation period. The total symptom score (TSS) was also recorded [13].

### Lactulose hydrogen breath test

Participants with a negative result in the lactose HBT were requested to return for a lactulose HBT within 1 week. Those patients, who did not excrete increased amounts of H_2_ (i.e., the peak of H_2_ breath excretion did not exceed the baseline level of 10 ppm) after oral administration of 10 g of lactulose in the lactulose HBT, were defined as hydrogen non-producers [14].

### Statistical analyses

All variables were expressed as mean ± s.d. or median and ranges as appropriate. The continuous variables were compared between LM and LI groups using the Student *t* test or the Mann-Whitney *U* test. Categorical variables were compared using a chi-square test. The statistical analysis was performed using SPSS 16.0 statistics software, and a *P* value < 0.05 was considered to be statistically significant. Sensitivity, specificity, and positive and negative predictive values were calculated using standard formulae [15].

## Results

### Description of the study cohort

A total of 115 patients with IBS-D who met the Rome III criteria were enrolled in this study. Six of the initial enrollees were excluded from the final analysis due to persistently high basal breath hydrogen. Ultimately, 109 patients with IBS-D and 50 healthy subjects were finally enrolled. The demographic and laboratory parameters of IBS-D patients and healthy controls are presented in Table 1.

**Table 1:** Comparison of the demographic and main clinical results of IBS-D patients and healthy subjects

	IBS-D patients (*n* = 109)	Healthy subjects (*n* = 50)	Statistics
Age (years)	36.0 ± 12.2	34.8 ± 13.3	ns (*t* = 0.565, *P* = 0.573)
Male/female	57/52	26/24	ns (*χ* ^2^ = 0.001, *P* = 0.973)
BMI	21.40 ± 2.83	21.27 ± 2.32	ns (*t* = 0.286, *P* = 0.775)
LM (%)	85 (82/96)	72 (34/47)	*χ* ^2^ = 3.522, *P* = 0.061
LI (%)	45 (43/96)	17 (8/47)	*χ* ^2^ = 9.684, *P* = 0.002
Hydrogen non-producers (%)	12 (13/109)	6 (3/50)	ns (*χ* ^2^ = 1.330, *P* = 0.249)
Maximum H_2_ excretion (ppm)	61 ± 55	66 ± 58	ns (*t* = 0.474, *P* = 0.636)
Total H_2_ excretion (ppm)	390 ± 328	448 ± 375	ns (*t* = 0.965, *P* = 0.336)

*IBS-D* diarrhea-predominant irritable bowel syndrome, *BMI* body mass index, *LM* lactose malabsorption, *LI* lactose intolerance, *ns* not significant

### IBS-D is associated with greater prevalence of LI

The prevalence of LM was high and comparable in both groups (IBS-D patients and healthy subjects). However, there was greater prevalence of LI in IBS-D patients when compared to healthy subjects (Table 1). There were 43 IBS-D patients and 8 healthy subjects present the symptoms related to LI. The TSS of LI in patients with IBS-D and healthy subjects was 7 (6–9) and 6 (5–7), respectively (*Z* = 1.351, *P* = 0.177). The severity of each symptom also showed no difference between the two groups (Table 2). In patients with IBS-D, symptoms indicating LI were also observed in two patients with a negative lactose HBT. Nevertheless, the prevalence of symptoms in patients with a positive or negative lactose HBT was significantly different (*χ* ^2^ = 4.707, *P* = 0.03). The maximum H_2_ excretion and total H_2_ excretion were not significantly different between IBS-D patients and control subjects (Table 1).

**Table 2:** Severity of symptoms related to LI in IBS-D patients (*n* = 43) and healthy subjects (*n* = 8)

Symptoms, median (quartiles)	IBS-D patients	Healthy subjects	*P* value
Abdominal pain	1 (0–2)	0 (0–1)	0.153
Bloating	2 (1–2)	1 (0–2)	0.052
Borborygmus	2 (1–3)	2 (2–3)	0.348
Diarrhea	3 (2–4)	3 (2–3)	0.141
TSS	7 (6–9)	6 (5–7)	0.177

*TSS* total symptom score, *IBS-D* diarrhea-predominant irritable bowel syndrome

### Self-reported milk intolerance is unreliable for diagnosing LI

In patients with IBS-D, excluding the hydrogen non-producers, the frequency of LI in self-reported milk intolerance patients was 25/47 (53%), whereas the frequency of LI in those without self-reported milk intolerance was 18/49 (37%). The sensitivity, specificity, and positive and negative predictive values of self-reported milk intolerance for detection of LI were 58, 58, 53, and 63%, respectively. Self-reporting of milk-intolerance is unreliable for diagnosing LI in this regard.

## Discussion

The prevalence of LM has substantial geographical variation and is related to ethnicity. The prevalence of LM detected in IBS-D and controls in our study confirmed the suspected high prevalence of LM in southern China. There was no significant difference in LM between IBS-D patients and healthy volunteers. LM seemed not to predispose individuals to IBS-D. Our finding was similar to that reported by Gupta et al. [4]. It reported that the prevalence of LM was 72% in IBS patients and 60% in healthy subjects. However, another study [10] in Norway showed a much lower prevalence of 4.1 and 3.8% in IBS patients and healthy subjects, respectively. It suggests that LM is widely variable throughout the world.

The relationship between LI and IBS remains unclear. Our study found that the prevalence of LI was higher in patients with IBS-D than in healthy subjects. It is consistent with findings that reported in other studies [16, 17]. There is no significant difference for symptoms about LI between IBS patients and healthy controls. Some previous studies did not differentiate LM from LI and reported a comparable prevalence of LI between IBS patients and healthy subjects [4]. Although LM is comparable between IBS-D and controls in our study, LI is more prevalent in the IBS-D patients. But the severity of symptoms related to LI showed no significant difference between the IBS-D patients and the healthy subjects.

Interestingly, two IBS-D patients were identified with negative lactose HBT but still exhibited symptoms of LI following lactose ingestion during the observation period. It indicates that LM and LI can become uncoupled or false negative results for HBT. In a meta-analysis [18], approximately 33–97% of the patients with a positive lactose HBT result reported symptoms following lactose ingestion. Additionally, 0–71% of the lactose absorbers reported symptoms [18]. The presence of symptoms after lactose ingestion was more strongly associated with a positive lactose HBT result in most studies [18].

One previous study postulated that colonic gas production, in particular hydrogen release, may be related to the symptoms and pathogenesis of IBS [19]. However, our study showed that the maximum and total hydrogen excretion in IBS-D patients and controls were comparable, which is in consistent with the results of others [4, 20]. But there is little evidence to suggest that colonic gas production relates to the etiology of IBS.

We found that self-reported milk intolerance has low sensitivity, specificity, and positive and negative predictive values with respect to diagnosis of LI. One previous study [13] also reported that the positive and negative predictive values of self-reported milk intolerance in detecting LI in IBS patients were as low as 75 and 31%, respectively. Hence, self-reported milk tolerance/intolerance in IBS patients is not reliable for diagnosing LI. A potential confounding factor is the presence of other ingredients in milk such as β-lactoglobulin and casein, which are known to be allergenic in some individuals [21]. However, our findings do not support a major role for self-reporting milk intolerance in the diagnosis of LI, especially in IBS patients.

A potential limitation of this study is the lack of methane measurements during the course of the study. Sulfur-metabolizing and methanogenic bacteria can consume hydrogen. Four percent of patients with LM could not be identified if hydrogen was measured without concomitant methane determination [22, 23]. However, another study found that almost half of the patients with constipation-predominant IBS are methane producers, while none of the patients with diarrhea-predominant IBS produce methane [24]. It seems that a measurement of methane production might not seriously affect the findings of this study. Nevertheless, it is still unclear whether methane production is different between IBS patients and healthy individuals. The influence of methane production remains controversial. Another potential limitation is that the result of lactose HBT could be influenced by bacteria in small intestine, which may result in intraluminal fermentation of lactose. Some studies also found that small intestinal bacterial overgrowth was associated with a subset of patients with IBS [4]. But in our previous study, we combined lactose HBT with scintigraphic scanning and proved small intestinal bacterial overgrowth had little impact on the interpretation of lactose HBT [25].

## Conclusion

In summary, our study found that the prevalence of LM is high in southern China and is comparable between patients with IBS-D and healthy subjects. But the frequency of LI is significantly higher in IBS-D patients than in healthy subjects. Self-reported milk intolerance is unreliable for diagnosing LI.
